# Serum iron as a biomarker for “penumbra freezing” in patients with acute ischemic stroke

**DOI:** 10.3389/fnins.2025.1764523

**Published:** 2026-01-12

**Authors:** Xianwen Zhang, Zhiyao Xu, Linyan Li, Huimin Deng, Qiang Zhou, Jianyu Liu, Hua Liu

**Affiliations:** 1 Department of Neurology, The Third People’s Hospital of Chengdu (Affiliated Hospital of Southwest Jiaotong University), College of Medicine, Southwest Jiaotong University, Chengdu, Sichuan, China; 2 Department of Clinical Medicine, North Sichuan Medical College, Nanchong, Sichuan, China; 3 Department of Neurology, The Affiliated Hospital of Southwest Jiaotong University, The Third People’s Hospital of Chengdu, Chengdu, Sichuan, China; 4 Medical Research Center, The Affiliated Hospital of Southwest Jiaotong University, The Third People’s Hospital of Chengdu, Chengdu, Sichuan, China

**Keywords:** acute ischemic stroke, biomarker, ferroptosis, penumbra freezing, serum iron

## Abstract

**Background:**

Identifying salvageable penumbra is crucial for revascularization in acute ischemic stroke (AIS) patients presenting beyond standard therapeutic windows. Dysregulated iron metabolism and ferroptosis play significant roles in the pathophysiology of cerebral ischemia. This study aimed to investigate serum iron as a biomarker for identifying the “penumbra freezing” (PF) phenomenon in AIS patients who present beyond the conventional time window.

**Methods:**

This study included patients with AIS presenting late (beyond 4.5-h treatment window). Participants were classified into PF and absence of PF (APF) groups based on CT perfusion and EXTEND trial criteria. Multivariable regression analyzed associations with PF, and diagnostic accuracy was evaluated via ROC curves.

**Results:**

A total of 141 AIS patients were finally included (Age: 71.16 ± 12.37, 54% male). Serum iron levels were significantly lower in the PF group compared with the APF group (8.59 ± 4.34 μmol/L vs. 11.54 ± 6.37 μmol/L, *p* = 0.04). The initial ROC analysis yielded an AUC of 0.63 (95% CI: 0.48–0.79, *p* = 0.09). After adjustment for confounders, the model’s AUC improved significantly to 0.76 (95% CI: 0.63–0.89, *p* < 0.01).

**Conclusion:**

Serum iron levels correlate with the PF phenomenon in AIS patients beyond the time window, potentially serving as an auxiliary biomarker to aid in identifying suitable candidates for delayed reperfusion therapy. This indicator offers convenient and rapid detection with clinical translation potential, though further validation in prospective, multicenter, large-scale studies remains necessary.

## 1 Introduction

Stroke refers to focal neurological impairment caused by acute cerebrovascular disease and remains one of the leading causes of disability and mortality worldwide. Ischemic stroke (IS), accounting for approximately 85% of all stroke cases, presents with acute onset and severe pathological consequences characterized by high incidence, mortality, and disability rates (Hilkens et al., 2024; Martin et al., 2025). The burden of stroke is particularly severe in China, where patients often experience substantial declines in quality of life, and families and society face heavy caregiving demands, making stroke an urgent public health challenge (GBD 2021 Stroke Risk Factor Collaborators, 2024).

Current guidelines recommend early reperfusion within the “time window” as the most effective strategy for salvaging the ischemic penumbra (Powers et al., 2019; Liu et al., 2023). Public education initiatives such as “Stroke 120” (Zhao and Liu, 2017), optimization of emergency medical response protocols (Royan et al., 2024), and establishment of stroke emergency networks (Ye et al., 2019) have collectively reduced prehospital delays. Moreover, advances in artificial intelligence have enabled rapid, automated quantification of infarct core and penumbra volumes, mitigating the limitations of subjective neuroimaging interpretation and reducing in-hospital delays (Wang et al., 2024). Nevertheless, the proportion of patients receiving timely reperfusion therapy remains limited (Tsivgoulis et al., 2023). Thus, developing convenient and reliable markers for early recognition of ischemic penumbra could assist clinicians in rapidly identifying salvageable tissue, further reducing door-to-needle time (DNT) and offering new approaches to extend the therapeutic window through “penumbra freezing (PF).”

Following cerebral ischemia, a cascade of biochemical reactions results in neuronal injury and cell death (Majumder, 2024; Poe et al., 2024). Among these, calcium overload plays a central role in glutamate excitotoxicity and oxidative stress-induced neuronal damage (Neves et al., 2023). Hyponatremia has also been shown to impair cerebral perfusion, worsen vascular injury, and negatively affect outcomes (Wang et al., 2021; Barkas et al., 2023). Recent animal studies indicate that iron ions promote ferroptosis and necroptotic apoptosis; moreover, ferroptosis inhibitors significantly reduce infarct size and mitigate ischemia–reperfusion injury (Du et al., 2024; Tuo and Lei, 2024; Tuo et al., 2017). Electrolyte testing—efficient, convenient, and cost-effective—is widely used clinically, yet few studies have explored their value in assessing the ischemic penumbra.

In clinical practice, many patients miss the opportunity for conventional thrombolysis due to delayed presentation, underscoring the need for novel assessment strategies beyond time-based criteria. This study collected data from 141 acute ischemic stroke (AIS) patients presenting 4.5-h after onset and classified them based on CT perfusion (CTP) results according to “tissue window” criteria (Liu et al., 2023). We aimed to explore the association between serum electrolytes and PF to provide an optimized strategy for expanding eligibility for standardized thrombolysis.

## 2 Materials and methods

### 2.1 Study population

A retrospective review was conducted on 141 AIS patients who presented beyond the standard 4.5-h treatment window at the Third People’s Hospital of Chengdu between January 2022 and September 2023. The inclusion criteria were: (1) diagnosis of AIS confirmed by imaging; (2) hospital presentation beyond the standard 4.5-h treatment window; (3) availability of complete CTP imaging and clinical data. Exclusion criteria were: (1) history of diseases known to significantly influence iron metabolism or systemic inflammation, including active infection, concurrent malignancy, or autoimmune/immune system disorders; (2) use of medications affecting iron levels (e.g., iron supplements, erythropoiesis-stimulating agents, long-term anticoagulants) within the past 3 months; (3) history of blood transfusion within the past 3 months; (4) pregnancy or lactation; (5) other types of stroke (hemorrhagic or transient ischemic attack); (6) incomplete or poor-quality imaging data. (7) diagnosed by imaging with posterior circulation infarction or lacunar infarction.

AIS diagnosis followed the 2019 Chinese Guidelines for the Diagnosis and Treatment of Acute Ischemic Stroke (Liu et al., 2020). Collected demographic variables included age, sex, hypertension, type 2 diabetes (T2D), smoking, alcohol consumption, body mass index (BMI), first-time onset, NIHSS, TOAST classification, Onset-to-door time and CTP findings.

Smoking was defined as daily consumption of ≥1 cigarette for ≥1 year (Kelly et al., 2008). Alcohol consumption referred to ≥12 standard drinks in the past year (Kelly et al., 2008). Hypertension and diabetes diagnoses followed WHO/ISH and ADA criteria (Wang, 2025; ElSayed et al., 2023). Hyperlipidemia followed the 2023 Chinese guidelines (Joint Committee on the Chinese Guidelines for Lipid Management, 2023).

According to the EXTEND trial, the determination of PF is based on the following CTP quantitative criteria: the ratio of the volume of the hypoperfused region (Tmax >6 s) to the volume of the infarct core (relative cerebral blood flow <30%) must be >1.2; the absolute volume difference between the two must be >10 mL; and the volume of the infarct core must be <70 mL. Failure to meet any of these criteria is defined as absence of PF (APF) (Ma et al., 2012).

All biochemical tests, including electrolytes and metabolic markers, were performed using fully automated analyzers (Beckman AU5800 and Mindray BC-6800plus). Test kits for electrolyte testing were supplied by Beckman Coulter. Blood samples were collected immediately upon hospital arrival, and all tests were completed within 1 hour after admission to the emergency department. All data were recorded by two staff members from the department and securely stored under strict confidentiality protocols.

Ethical approval for this study involving human participants was obtained the Ethics Committee of the Third People’s Hospital of Chengdu and conducted in accordance with the ethical principles outlined in the Declaration of Helsinki. Written informed consent was acquired from all participants; for those unable to sign personally, the informed consent procedures were completed by their immediate family members acting as legal representatives.

### 2.2 Statistical analysis

Continuous variables were tested for normality using the Shapiro–Wilk test. Normally distributed data are presented as mean ± standard deviation and compared between groups using the independent samples *t*-test. Non-normally distributed data are presented as median (interquartile range) and compared using the Mann–Whitney *U* test. Categorical variables are presented as number (percentage) and compared using the Chi-square test or Fisher’s exact test, as appropriate.

Variables for inclusion in the multivariable logistic regression model were selected based on a combination of clinical relevance and statistical criteria from univariate analyses. All variables with a *p*-value < 0.10 in univariate analyses, along with key demographic factors (e.g., age, sex) deemed clinically important, were entered into the initial model. A backward stepwise elimination procedure (with a retention criterion of *p* < 0.05) was then used to identify independent factors associated with PF. The model’s goodness-of-fit was assessed using the Hosmer-Lemeshow test. Results of the final logistic regression model are presented as adjusted odds ratios (aORs) with their corresponding 95% confidence intervals (95% CIs) and *p*-values.

Receiver Operating Characteristic (ROC) curve analysis was conducted to evaluate the diagnostic performance of serum iron for predicting PF. The optimal cutoff value for serum iron was determined by maximizing Youden’s index (J = sensitivity + specificity − 1). The sensitivity, specificity, and the area under the curve (AUC) at this optimal cutoff are reported.

A two-tailed *p*-value < 0.05 was considered statistically significant for all analyses. All statistical analyses were performed using Graphpad Prism software (version 10.1.2).

## 3 Results

### 3.1 Demographic and clinical characteristics

**Figure 1:**
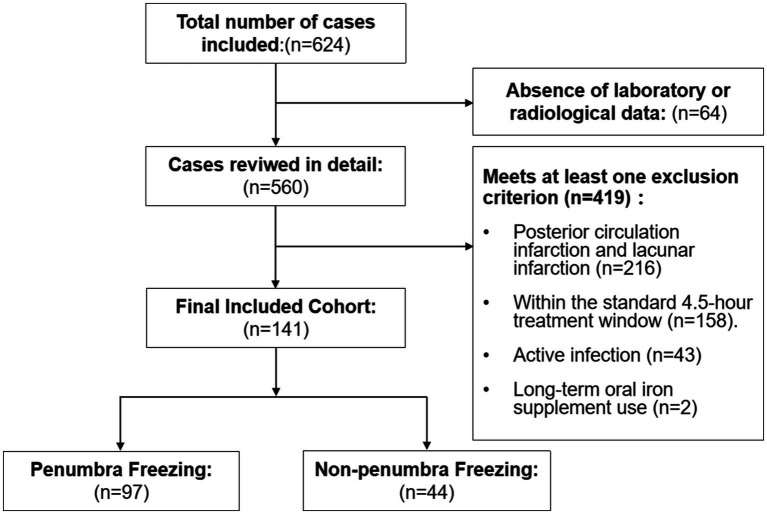
Flowchart of patients screening.

A final cohort of 141 eligible AIS patients was included in the analysis (Figure 1). No significant differences were found in age, hypertension, diabetes, first onset, BMI, smoking, or alcohol consumption between PF and APF groups. However, the multivariate analysis indicated associations between PF and gender, hyperlipidemia (Table 1).

**Table 1:** Baseline characteristics.

Variable	Total	PF	APF	Unadjusted OR (95% CI)	Adjusted OR (95% CI)	*p*
Demographics						
Age, years	71.16 (12.37)	70.89 (12.93)	71.67 (11.51)	1.00 (0.95, 1.04)	1.00 (0.93, 1.08)	0.81
Sex, male, *n* (%)	76 (53.9)	59 (60.8)	17 (38.6)	0.41 (0.20, 0.84)	0.43 (0.13, 1.26)	**0.02**
BMI, kg/m^2^	22.76 (3.92)	22.88 (4.37)	22.55 (3)	1.00 (0.90, 1.19)	1.16 (0.92, 1.56)	0.24
Risk factors						
Hypertension, *n* (%)	102 (72.3)	68 (70.1)	34 (77.3)	1.45 (0.63, 3.32)	1.23 (0.18, 7.80)	0.82
Diabetes, *n* (%)	41 (29.1)	29 (29.9)	12 (27.3)	0.88 (0.40, 1.94)	0.40 (0.07, 1.82)	0.26
Hyperlipidemia, *n* (%)	55 (39.0)	34 (37.4)	21 (53.8)	1.96 (0.92, 4.18)	0.24 (0.07, 0.81)	**0.04**
First onset, *n* (%)	114 (80.9)	81 (83.5)	33 (75.0)	0.59 (0.25, 1.41)	0.61 (0.25, 1.78)	0.46
Smoking, *n* (%)	51 (36.2)	38 (40.0)	13 (29.5)	0.63 (0.29, 1.35)	1.11 (0.45, 5.33)	0.85
Drinking, *n* (%)	30 (21.3)	23 (24.2)	7 (15.9)	0.59 (0.23, 1.51)	1.34 (0.18, 3.65)	0.93
Stroke characteristics						
NIHSS, median (IQR)	12 (5.5)	12 (6.0)	10.5 (3.4)	0.99 (0.93, 1.06)	0.89 (0.79, 1.00)	0.06
Onset-to-door time, min, median (IQR)	390 (300)	390.0 (310.5)	420.0 (282.0)	0.98 (0.91, 1.06)	0.96 (0.84, 1.09)	0.54
TOAST, LAA, *n* (%)	43 (64.2)	28 (63.6)	15 (65.2)	0.91 (0.31, 2.55)	0.68 (0.12, 3.35)	0.64

Data are means (SD) or median (IQR), PF, penumbra freezing; APF, absence of penumbra freezing; CI: 95%, confidence interval; BMI, body mass index [kg/m^2^]. Bold values indicate statistically significant differences (*p* < 0.05).

### 3.2 Laboratory characteristics

Most laboratory indicators showed no significant group differences. However, higher ALB were associated with PF (Table 2).

**Table 2:** Laboratory parameters.

Variable	Total	PF	APF	Unadjusted OR (95% CI)	Adjusted OR (95% CI)	*p*
Full blood count						
WBC	9.67 (4.26)	10.17(4.45)	8.72 (3.77)	1.09 (0.96, 1.27)	1.28 (1.01, 1.73)	0.07
PLT	189.94 (72.54)	185.68 (77.04)	197.86 (64.25)	1.00 (0.99, 1.01)	1.01 (0.99, 1.02)	0.30
Liver function tests						
ALB	37.6 (5.5)	36.65 (−5.89)	39.29 (4.34)	0.91 (0.81, 1.00)	0.84 (0.70, 0.99)	**0.05**
AST	35.71 (42.63)	37.5 (51.56)	32.73 (21.33)	1.00 (1.00, 1.02)	0.97 (0.93, 1.09)	0.10
ALT	28.46 (24.1)	29.09 (25.71)	27.34 (21.45)	1.00 (0.98, 1.03)	1.00 (0.96, 1.05)	0.86
Renal function tests						
BUN	7.19 (5.61)	8.05 (6.71)	5.64 (2.05)	1.16 (1.00, 1.45)	1.43 (0.97, 2.23)	0.10
CR	91.34 (73.15)	101.35 (87.59)	73.07 (26.99)	1.02 (1.00, 1.04)	1.02 (0.98, 1.07)	0.38
UA	331.15 (128.72)	345.55 (132.05)	304.84 (120.75)	1.00 (1.00, 1.01)	1.00 (1.00, 1.08)	0.79
eGFR	94.06 (33.51)	81.33 (32.79)	89.06 (34.96)	0.99 (0.98, 1.01)	1.00 (0.97, 1.01)	0.72
Cardiac function						
BNP	325.16 (509.19)	362.26 (560.19)	243.01 (377.88)	1.00 (1.00, 1.00)	1.00 (1.00, 1.00)	0.36

Data are means (SD), WBC, white blood cell [10^9^/L]; PLT, blood platelet [×10^9^/L]; ALB, albumin [g/L]; AST, aspartate aminotransferase [U/L]; ALT, alanine transaminase [U/L]; BUN, blood urea nitrogen [mmol/L]; CR, serum creatinine [μmol/L]; UA, uric acid [μmol/L]; BNP, brain natriuretic peptide [pmol/L]; eGFR, estimated glomerular filtration rate [mL/min/1.73m^2^]. Bold values indicate statistically significant differences (*p* < 0.05).

### 3.3 Association between serum iron and ischemic penumbra

**Figure 2:**
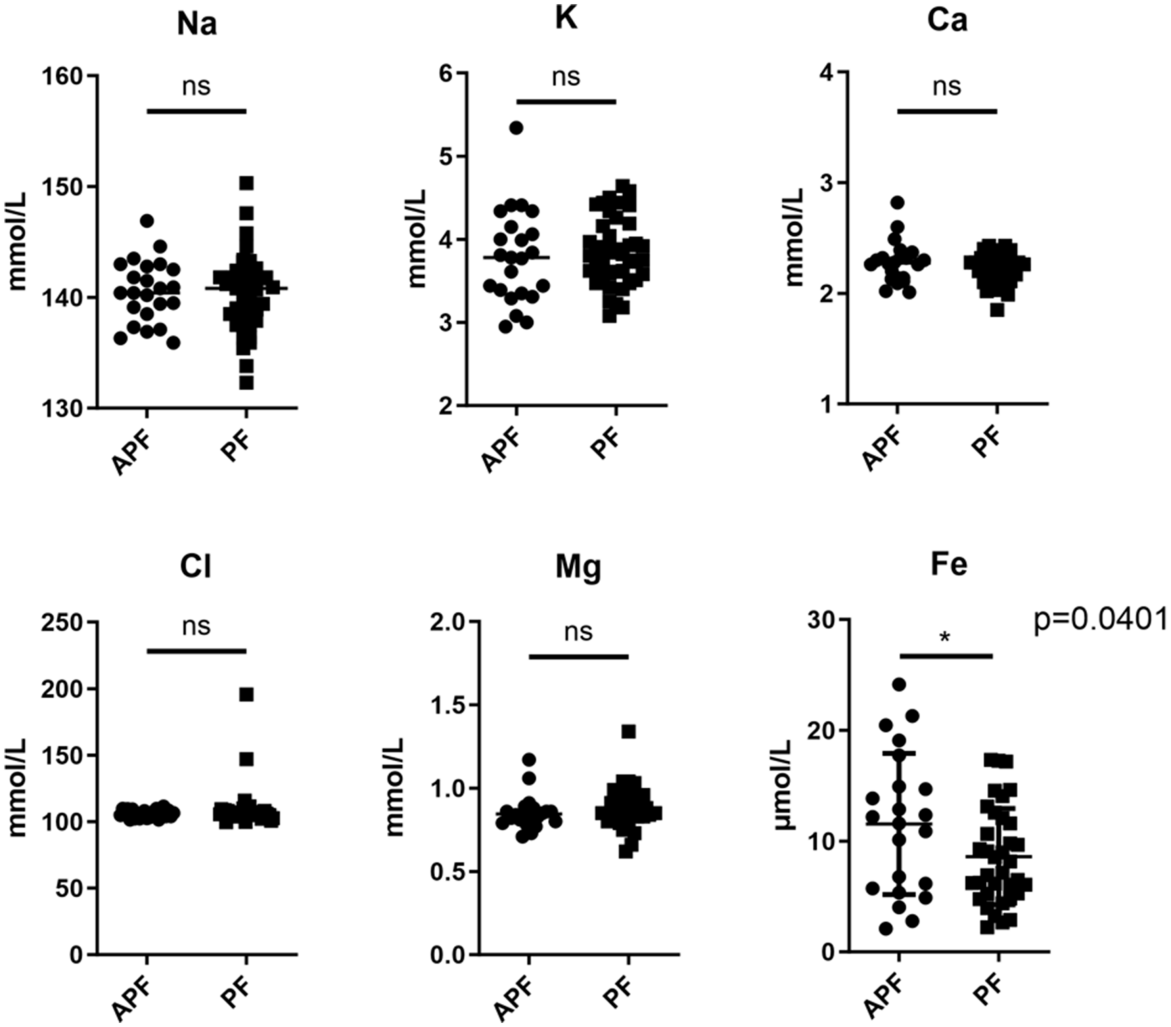
Serum electrolyte discrepancy analysis. APF, absence of “penumbra freezing”; PF, “penumbra freezing.” *p < 0.05.

Serum iron levels were significantly lower in the PF group (8.59 ± 4.34 μmol/L) compared with the APF group (11.54 ± 6.37 μmol/L, *p* = 0.04). Other electrolytes showed no significant differences (Figure 2).

**Figure 3:**
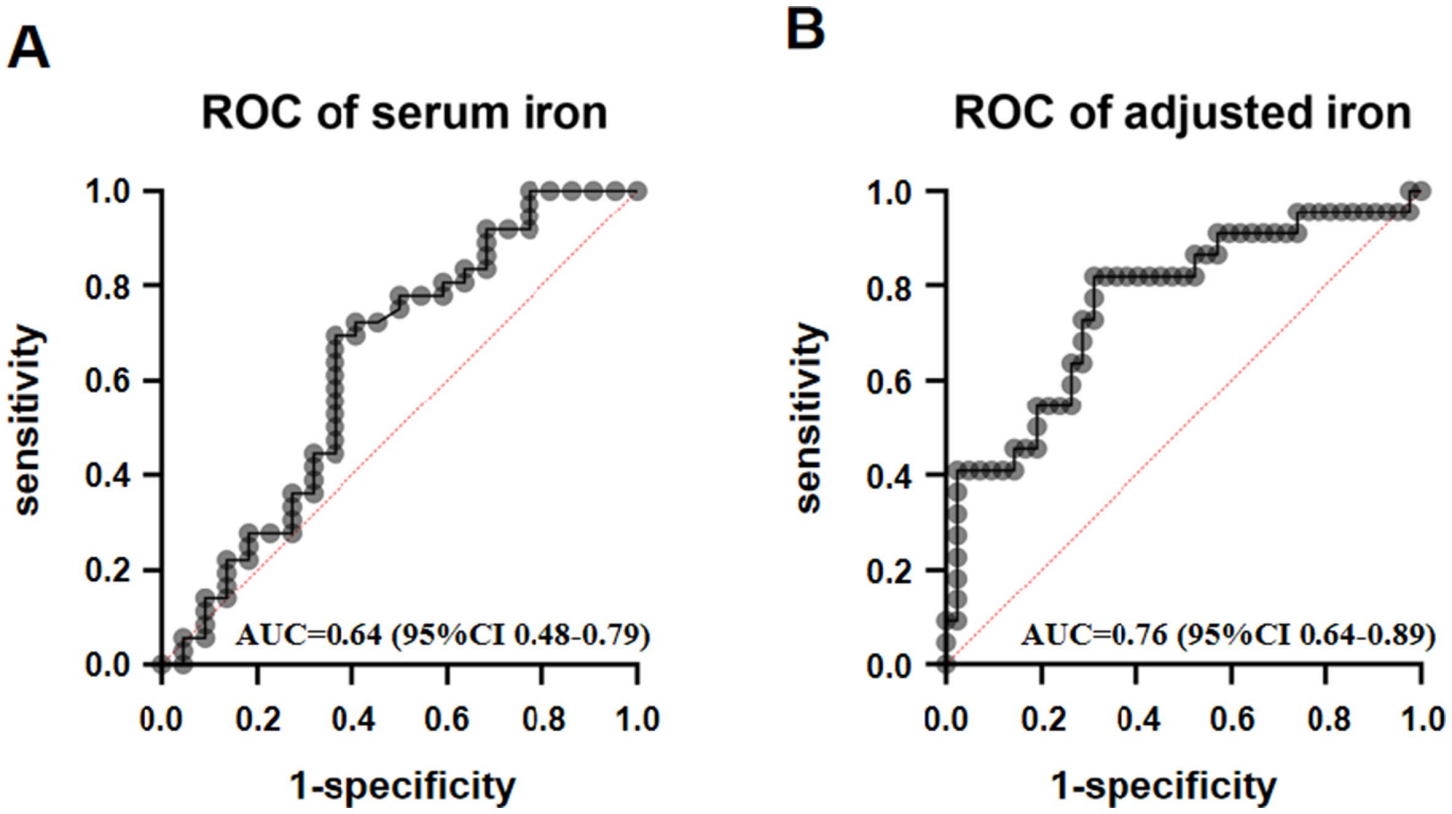
ROC curve of serum iron in “penumbra freezing.” **(A)** shows the ROC curve for serum iron, while **(B)** shows the ROC curve for adjusted serum iron, with correction factors including age, gender, BMI, hypertension, diabetes, smoking, and alcohol consumption.

The unadjusted analysis revealed that serum iron had an area under the curve (AUC) of 0.64 (95% CI: 0.48–0.79; *p* = 0.08) for predicting PF. The optimal cutoff, determined by maximizing Youden’s index, was 9.96 μmol/L, which corresponded to a sensitivity of 0.69 and a specificity of 0.63. After adjusting for potential confounders, the AUC increased to 0.76 (95% CI: 0.64–0.89; *p* < 0.01), indicating a statistically significant improvement in diagnostic performance (Figure 3).

## 4 Discussion

Our study found significant differences in serum iron levels between the PF group and the APF group, with the PF group showing markedly lower levels than the APF group. After adjusting for confounding factors, the diagnostic accuracy of serum iron improved (AUC increased to 0.76), indicating moderate discriminatory ability and suggesting its potential as a biomarker. This marker may aid in identifying the penumbral zone where salvage is still possible beyond the standard time window. Serum iron testing is convenient, rapid, and inexpensive (Pfeiffer and Looker, 2017), making it suitable for integration into emergency stroke workflows, including mobile stroke units and point-of-care testing (POCT) (Helwig et al., 2019; Dabla and Dabas, 2025). Combined with clinical assessment and neuroimaging studies, serum iron assessment may allow rapid preliminary identification of PF candidates, helping reduce DNT and potentially improve outcomes (Liu et al., 2023; Baskar et al., 2021).

The concept of “PF” has garnered increasing attention, with interventions such as hyperbaric oxygen (van der Worp et al., 2007), hypothermia (Macleod et al., 2010), remote ischemic conditioning (Chen et al., 2022; Blauenfeldt et al., 2023), and molecular targets like CircOGDH (Liu et al., 2022) showing promise. Iron plays a central role in ferroptosis, a lipid peroxidation-driven cell death pathway implicated in ischemic neuronal death (Guo et al., 2023; Hu et al., 2024). Imbalanced iron metabolism after ischemia leads to oxidative stress, inflammation, and cell death via ferroptosis and necroptosis (Zhang et al., 2024; He et al., 2024; Zhou et al., 2021). Our finding that lower serum iron is associated with PF aligns with the hypothesis that iron dysregulation contributes to penumbral viability.

Serum iron primarily exists as ferric iron ions bound to transferrin to form complexes. These complexes, along with a small fraction of ferrous ions, can bind to corresponding receptors (TFR1, DMT1) on the endothelial cell membranes of the blood–brain barrier and enter brain tissue via endocytosis (Qian and Ke, 2019; Ryan et al., 2018). Following ischemic stroke, compromised blood–brain barrier integrity allows both transferrin-bound iron and free iron to passively diffuse into brain tissue, leading to decreased serum iron levels. Dysregulation of hepcidin (Billesbølle et al., 2020), dysfunction of iron export proteins (Qian and Ke, 2019), and Blood–Brain Barrier (BBB) impairment can synergistically lead to cerebral iron deposition, thereby triggering key mechanisms of ferroptosis (Ferreira et al., 2019; Liu et al., 2024). Another critical mechanism involves iron ions catalyzing the Fenton reaction of hydrogen peroxide, generating highly reactive hydroxyl radicals that drive lipid peroxidation injury (Hu et al., 2024; Zhang et al., 2024). Concurrently, iron-related pathways may serve as pivotal junctions linking ferroptosis and necrotic apoptosis, accelerating cell death within the penumbra and undermining the maintenance of the “PF” (Gong et al., 2023). Lower serum iron levels imply a reduction in the total iron available to brain tissue, indirectly contributing to the maintenance of the ischemic penumbra. The association between serum iron levels and ischemic penumbra status may involve mechanisms such as blood–brain barrier disruption, iron transport abnormalities, ferritin dysfunction, and intracellular iron accumulation. Further mechanistic investigation of these pathways is urgently needed.

Meanwhile, multivariate analysis in our study revealed associations between PF and gender, hyperlipidemia. Potential mechanisms may involve the potent anti-inflammatory effects of progesterone and estrogen demonstrate protective actions against ischemic injury, potentially explaining gender differences in PF risk (Tamtaji et al., 2025). Dyslipidemia may exacerbate oxidative stress and inflammatory responses in the vascular endothelium, synergizing with ferroptosis pathways to jointly influence penumbra fate (Guo et al., 2023; Achón Buil and Rust, 2023; Zechariah et al., 2013). These factors may interact with iron metabolism to form a network influencing PF, which warrants further investigation.

This study primarily focused on biomarkers of PF and did not perform correlation analyses with clinical functional outcomes as the primary endpoint, which represents a limitation. However, based on existing literature, it is reasonable to hypothesize that earlier identification of PF patients through serum iron levels could significantly reduce prehospital treatment time. If successful reperfusion therapy is achieved, patients may experience improved neurological recovery (Liu et al., 2020; Baskar et al., 2021; Ciacciarelli et al., 2025). A core objective for future research is to explore whether interventions targeting iron metabolism can protect the penumbra and improve outcomes.

Our study is a single-center, retrospective case–control investigation with inherent limitations. The inclusion of patients exhibits geographic and healthcare resource specificity, and the relatively small sample size (*n* = 141) may introduce selection bias, limiting statistical power and affecting the generalizability of results to broader populations. The imbalance in case numbers between the PF and APF groups (97 vs. 44 cases) may have affected the stability of diagnostic performance metrics and threshold setting. The inclusion variables did not account for underlying diseases, medications affecting serum iron levels (such as iron supplements, antibiotics, proton pump inhibitors, antiplatelet agents, etc.), or other iron metabolism-related indicators, which may represent potential confounding factors. We plan to pursue more comprehensive and detailed investigations in a multicenter, large-sample, and more balanced cohort. This will involve more systematic collection and control of potential confounding factors, alongside mechanism studies to elucidate the causal relationship between serum iron and the ischemic penumbra.

Despite multivariate adjustments and sensitivity analyses, the possibility of residual confounding cannot be entirely ruled out. This study was observational in design, and serum iron levels were not randomly assigned. While we adjusted for multiple known confounders, including age, major vascular risk factors, and stroke severity (NIHSS), and demonstrated the robustness of our findings, there may be other unmeasured or imprecisely measured factors (e.g., detailed dietary iron intake, specific inflammatory markers, genetic background) that could influence both baseline serum iron and the fate of the ischemic penumbra. These factors might act as potential confounders, partly explaining the observed association. Therefore, the present findings should be interpreted as suggesting a biologically plausible association rather than establishing definitive causality. Future prospective studies particularly those incorporating more comprehensive baseline measurements and potentially employing methods such as Mendelian randomization to better control for confounding, are essential to confirm a causal relationship.

## 5 Conclusion

This study indicates that lower serum iron levels correlate with the PF phenomenon in AIS patients beyond the time window and exhibit moderate discriminatory ability. Serum iron holds promise as a potential adjunct biomarker to aid in identifying patients who may be suitable for delayed reperfusion therapy. However, these findings require further validation in prospective, large-scale, multicenter cohorts, and exploration of its value in clinical decision-making models integrating multimodal imaging.

## Data Availability

The raw data supporting the conclusions of this article will be made available by the authors, without undue reservation.
